# Factors influencing stress, anxiety, and depression among Iranian pregnant women: the role of sexual distress and genital self-image

**DOI:** 10.1186/s12884-021-03575-1

**Published:** 2021-01-26

**Authors:** Afsaneh Keramat, Mina Malary, Mahmood Moosazadeh, Nastaran Bagherian, Mohammad-Reza Rajabi-Shakib

**Affiliations:** 1 Department of Reproductive Health and Midwifery, School of Nursing and Midwifery, Shahroud University of Medical Sciences, Shahroud, Iran; 2 Student Research Committee, School of Nursing and Midwifery, Shahroud University of Medical Sciences, Haft-e Tir Square, Po Box: 7394736147, Shahroud, Iran; 3 Health Sciences Research Center, Faculty of Health, Mazandaran University of Medical Sciences, Sari, Iran; 4 Gastrointestinal Cancer Research Center, Non-communicable Diseases Institute, Mazandaran University of Medical Sciences, Sari, Iran; 5 Midwifery Counseling, Mazandaran University of Medical Sciences, Sari, Iran; 6 General Practitioner, Independent Researcher, Tehran, Iran

**Keywords:** Pregnancy, Depression, Anxiety, Stress, Genital self-image, Sexual distress

## Abstract

**Background:**

Pregnancy is a unique period with the increased likelihood of psychological changes and emotional disturbances such as depression, anxiety, and stress. In this study, we investigated the factors influencing depression, anxiety, and stress in pregnancy and identify their associations with Sexual Distress (SD) and Genital Self-Image (GSI).

**Methods:**

This was a descriptive, correlational, cross-sectional study performed using the two-stage cluster sampling method between September 2019 and January 2020. Overall, 295 pregnant women completed a demographics and obstetric information checklist, Depression Anxiety and Stress Scale-21 (DASS-21), Female Genital Self-Image Scale (FGSI), and Female Sexual Distress Scale-Revised (FSDS-R).

**Results:**

Analysis of Variance (ANOVA) showed significant differences in the mean scores of SD between the groups with varying degrees of depression, anxiety, and stress (*P* <  0.001) and in the mean score of GSI between the groups with varying degrees of depression (*P* = 0.01) and anxiety (*P* <  0.001). In multivariate linear regression analysis, higher (worse) depression, anxiety, and stress scores were found in women with more advanced age and higher SD scores; however, these scores were lower (better) in those with increased gestational age. Lower depression and anxiety scores were associated with moderate satisfaction with income, moderate satisfaction with BI in pregnancy, and lower stress and depression scores were linked to planned pregnancy. Higher (better) GSI score was a predictor of lower depression score, complication in a previous pregnancy was a predictor of higher stress score, and finally, fear of fetal abortion and being a housewife were predictors of a higher anxiety score.

**Conclusion:**

Various factors contribute to the development of antenatal depression, anxiety, and stress. A positive correlation was found between SD and the severity of depression, anxiety, and stress, while a negative correlation was noted between GSI and the severity of depression and anxiety. Therefore, raising awareness regarding SD and GSI through screening and counseling sessions can have beneficial effects for mothers and their fetuses.

**Supplementary Information:**

The online version contains supplementary material available at 10.1186/s12884-021-03575-1.

## Background

Pregnancy is one of the most critical periods with considerable changes in women’s physical, mental, and sexual states [1]. During this period, vulnerability to emotional and psychological conditions such as depression, anxiety, stress, and psychosis is increased, which can lead to maternal and fetal adverse consequences [2].

The rates of the common mental disorders during pregnancy such as depression and anxiety range from 4 to 25% in different studies [3, 4]. In one study, the prevalence rates of prenatal stress, anxiety, and depression during the first weeks of pregnancy were reported 91.86, 15.04 and 5.19%, respectively [5]. The findings of an Iranian study also reported the rates of depression, anxiety, and stress to be 31.7, 32.5, and 49.1%, respectively [6]. The combination of maternal depression, stress, and anxiety can cause preterm labor, preeclampsia, and fetal neurodevelopmental problems [7].

Many factors seem to affect the mental state of pregnant women. In a study by Tang et al., it was reported that anxiety, low social support, and poor and/or moderate-level family care were the risk factors for prenatal depression. The risk factors for prenatal anxiety included unemployment, primiparity, stress, depression, and low social support. In addition, unemployment, anxiety, and low- and moderate-level social support were found to be associated with the development of prenatal stress [5]. There are limited studies on the effect of sexual distress (SD) and genital self- image (GSI) on prenatal stress, anxiety, and depression.

SD is considered as a state of experiencing negative emotions such as embarrassment, blaming, frustration, anxiety, fear, and anger in one’s sexual life [8]. Decreased sexual desire [9], worry, fear of harm due to sexual activity [10], notable changes in body image (BI) and appearance [11], and poor adaptation to sexual changes and parental role [12] can be considered as the main factors instigating SD during pregnancy. In Canadian and American population-based studies of pregnant women, 40% were found to experience SD during pregnancy [13]. However, it is unclear whether increased SD can lead to development of anxiety, depression, or stress during this period.

GSI is a subcategory of BI [14], which is defined as one’s perception and experience of genitals, such as appearance, odor, and functionality [15]. During pregnancy several alterations occur throughout the body, including weight gain and skin changes [16]. In the study by Earle, pregnant women expressed concerns regarding how they would look as their pregnancy advances, which parts of their body would change, and how hard it would be to get back to their pre-gestational body shape [17]. The genital area is one of the most important areas of the body that undergoes different changes during pregnancy [18]. It has been found that BI perception levels decline during pregnancy [16]. Some studies have investigated the relationship between BI and prenatal mental disorders such as depression [19, 20], which showed that poor BI during the third trimester of pregnancy is a risk factor for post-partum depression [21]. However, pregnant women’s perceptions about their genitals and its relationship with mental disorders such as depression, anxiety, and stress during pregnancy is still uncertain.

Various factors are known to cause or exacerbate depression, anxiety, and stress during pregnancy. Thus, promoting awareness regarding the risk factors for depression, anxiety, and stress during pregnancy based on regional and cultural contexts is critical in planning and implementing prenatal care programs because of the unique characteristics of this period.

Therefore, we sought to determine the associations between SD and GSI and depression, anxiety, and stress and to identify the other factors (including demographic, obstetric, and psychological factors addressed in previous studies) influencing the development of antenatal depression, anxiety, and stress in an Iranian population.

## Methods

### Study population and design

This cross-sectional survey was performed between September 2019 and January 2020 using the two-stage cluster sampling method in Amol, north of Iran. It must be noted that this study is presented according to Strengthening the Reporting of Observational studies in Epidemiology (STROBE) guidelines [22].

First, four healthcare centers in four regions of Amol city (north, south, east and west) were randomly selected. Second, pregnant women were selected by the systematic sampling method from the Department of Midwifery of each center. The sample size from each center was determined based on the probability of selection in proportion to population size. Then, the pregnant women meeting the inclusion criteria were explained the purpose and nature of the study and signed an informed consent form.

The inclusion criteria consisted of healthy pregnant women, women with singleton pregnancy in all trimesters, living with the spouse at the time of recruitment, and willingness to participate in the study. The exclusion criteria were women with contraindication for sexual intercourse (for any reason), women with any medical illness and/or complication in the current pregnancy, and women fertilized via assisted reproductive techniques.

### Sample size

In a study of pregnant women, the mean and standard deviation of anxiety score using the Depression, Anxiety and Stress scale (DASS-21) in Iran was 4.23 (SD = 4.23) [23]. With an estimated precision of 50% (d = 0.5), confidence level of 95% (α) (Z = 1.96), and an attrition rate of 15%, the final sample size was estimated at 323 pregnant women.

### Measures

Four instruments were applied for data collection, including Depression Anxiety and Stress Scale-21 (DASS-21), Female Sexual Distress Scale-Revised (FSDS-R), Female Genital Self-Image Scale (FGSI), and a socio-demographics checklist for sample characterization.

### Socio-demographics checklist

This checklist was composed of objective questions designed by the researchers and contained socio-demographic (i.e., age, duration of marriage, women’s education, women’s occupation, satisfaction with income), obstetric (parity, planned pregnancy, history of abortion, complications in previous pregnancy, gestational age, and fear of fetal abortion) and psychological (satisfaction with foreplay and satisfaction with BI) questions. The checklist developed for this study is provided as Additional file 1.

### Depression anxiety and stress Scale-21 item (DASS-21)

The validated version of the short form of Depression Anxiety Stress Scale (DASS), the DASS-21, was developed by P. F. Lovibond and S. H. Lovibond (1995) to reduce administration time and has been used widely among clinical samples to screen for symptoms of different levels of depression, anxiety and stress [24, 25]. Seven items are assigned for the evaluation of depression, anxiety and stress. Each item is scored from never (0) to very high (3), with higher scores indicating greater levels of depression, anxiety and stress. In an English study, the Cronbach’s alpha was reported 0.95 for depression, 0.90 for anxiety, 0.93 for stress, and 0.97 for the overall scale [26]. The reliability coefficients of the Persian version of the DASS-21 were reported 94, 92, and 82% for the domains of depression, anxiety, and stress, respectively [27], indicating its acceptable reliability and validity among Iranian samples. The scoring method is presented in Table 1 [28].

**Table 1:** Method of scoring the severity of depression, anxiety and stress

Score	Depression	Anxiety	Stress
Normal	0–9	0–7	0–14
Mild	10–13	8–9	15–18
Moderate	14–20	10–14	19–25
Sever	21–27	15–19	26–33
Very sever	≥28	≥20	≥34

### Female sexual distress scale-revised (FSDS-R)

Before 2008, the FSDS became the most extensively validated and widely used scale for assessing sexuality-related distress in women. In 2008, a revised version of the FSDS, the FSDS-R, was developed with the addition of a 13th item, offering the tool increased sensitivity specificity [8]. Both of these scales are widely accepted and translated into different languages and validated in various cultures and populations [8, 29–31]. All 13 items of FSDS-R are rated on a five-point Likert-type scale ranging from 0 (never) to 4 (always). The total score can be computed by adding all item scores and ranges from 0 to 52, with a score of 11 or higher score indicating SD in women during the past month. The original version of the FSDS-R showed high internal consistency with Cronbach’s alpha values ranging from α = 0.87 to α = 0.93 and high test–retest reliability (ranging from *r* = 0.74 to *r* = 0.86) [31]. Also, the internal consistency and reliability of the Persian version of FSDS-R were established by Azimi et al. to be more than 0.70 [30].

### Female genital self-image scale (FGSI)

The FGSIS was developed by Herbenick et al. (2010) to evaluate women’ feelings and beliefs about their genitals [32]. It is composed of seven items, and each item is rated on a 4-point Likert scale ranging from 1 = strongly disagree to 4 = strongly agree. The range of scores is from 7 to 28, with higher scores indicating a more positive GSI. The FGSIS has been reported to have high reliability and good validity [33]. In the study conducted by Felix et al., Cronbach’s Alpha was reported 0.81 for this instrument, which indicates its high reliability [34]. The Cronbach’s alpha and test–retest reliability of the Persian version of FGSIS was demonstrated 86%, indicating a good degree of internal consistency [35].

### Statistical analysis

Descriptive statistics were used to analyze the dependent (depression, anxiety and Stress) and independent (i.e., women’s socio-demographic, obstetric, and psychological information) variables.

Analysis of Variance (ANOVA) was used for comparing the mean scores of SD and GSI between the groups with different degrees of depression, anxiety, and stress. In addition, if necessary, the Post Hoc Tukey’s test was run.

Firstly, the factors associated with depression, anxiety, and stress were identified using the univariate linear regression. A *P*-value of lower than 0.25 in the univariate linear regression analysis was adopted as representing the critical level for the selection of variables. Finally, multivariate linear regression analysis was used to assess the relationships between the dependent and independent variables. Statistical significance was set at *P*-value less than 0.05.

## Results

### Participants’ characteristics

All the participants were aged 18–40 years (mean = 28.3) with a mean gestational age of 25.08 weeks. The mean scores of depression, anxiety, and stress were 8.43 ± 7.40, 10.01 ± 7.61, and 13.13 ± 8.82, respectively. Most (76.3%) of the participants had planned pregnancy, and almost half of them (52.9) were multiparous. Furthermore, most (85.0) of the participants were housewives, 39% had a college degree or above, 50% had a high school education, and 10% had a secondary school degree or below.

The data regarding the other variables such as duration of marriage, satisfaction with income, history of abortion, complications in previous pregnancy, fear of fetal abortion, and satisfaction with foreplay and BI is shown in Table 2.

**Table 2:** Descriptive statistics for all participant characteristics (*n* = 295)

Participant characteristics	N (%)	Mean ± SD (range)
**Demographic factors**		
**Age(y)**	–	28.3 ± 5.27 (18–40)
**Duration of marriage(y)**	–	6.04 ± 4.75 (1–22)
**Education level**		
Primary/secondary school	31 (10.5)	–
High school	149 (50.5)	–
Undergraduate/postgraduate	115 (39.0)	–
**Women occupation**		
Working	44 (14.9)	–
Housewife	251 (85.0)	–
**Satisfaction with income**		
Low	73 (24.7)	–
Moderate	212 (71.9)	–
High	10 (3.4)	–
**Obstetric factors**		
**Parity**		
Primiparous	139 (47.1)	–
Multiparous	156 (52.9)	–
**Planned pregnancy**		
Yes	255 (76.3)	–
No	70 (23.7)	–
**History of abortion**		
Yes	65 (22)	–
No	230 (78)	–
**Complication in previous pregnancy**		
Yes	32 (10.8)	–
No	263 (89.2)	–
**Fear of fetal abortion**		
Yes	145 (49.2)	–
No	150 (50.8)	–
**Gestational age**	–	25.08 ± 9.09 (4–40)
**Psychological factors**		
**Satisfaction with BI in pregnancy**		
Low	95 (32.5)	–
Moderate	171 (58.0)	–
High	29 (9.8)	–
**Satisfaction with foreplay**		
Yes	267 (90.5)	–
No	28 (9.5)	–
**Sexual distress**	–	5.55 ± 6.56 (0–52)
**Genital Self-Image**	–	19.98 ± 3.97 (7–28)
**Depression**	–	8.43 ± 7.40 (0–36)
**Anxiety**	–	10.01 ± 7.61 (0–40)
**Stress**	–	13.13 ± 8.82 (0–42)

### Effect of SD and GSI on depression, anxiety, and stress

One-way ANOVA showed significant differences in the average score of SD among the groups with varying degrees of depression, anxiety, and stress. As the SD score increased, the intensity of each of the variables of depression, anxiety, and stress also raised (*p* <  0.001). Moreover, the mean score of GSI had a significant negative association with the degrees of depression (*p* = 0.011) and anxiety (*p* <  0.001). In other words, decreased mean score of GSI (poor GSI) was associated with increased severity of depression and anxiety (Table 3).

**Table 3:** The relationship between GSI, SD and stress, anxiety, depression

Variables	Categories	n (%)	Genital self-image			Sexual distress		
			Mean (SD)	***p***-value	ANOVA (F)	Mean (SD)	***p***-value	ANOVA (F)
**Stress**	Normal	182(61.7)	20.16 (3.8)	0.24	F = 1.36	3.97 (4.2)	< 0.001	F = 14.50
	Mild	42(14.2)	20.55 (3.1)			5.46 (5.5)		
	Moderate	38(12.9)	19.29 (3.6)			7.66 (10.9)		
	Sever	26(8.8)	18.65 (4.8)			11.19 (6.7)		
	Very sever	7(2.4)	20.57 (3.7)			15.29 (8.8)		
**Anxiety**	Normal	117(39.7)	20.16 (3.8)	< 0.001	F = 5.33	3.50 (6.37)	< 0.001	F = 11.81
	Mild	32(10.8)	20.55 (3.1)			4.53 (4.3)		
	Moderate	77(26.1)	19.29 (4.6)			6.10 (5.5)		
	Sever	38(12.9)	18.65 (4.8)			6.55 (6.01)		
	Very sever	31(10.5)	20.57 (3.7)			11.74 (8.1)		
**Depression**	Normal	173(58.6)	20.60 (4.0)	0.011	F = 3.34	3.83 (5.6)	< 0.001	F = 14.2
	Mild	58(19.7)	19.41 (3.1)			6.79 (7.0)		
	Moderate	41(13.9)	19.27 (3.9)			6.76 (5.5)		
	Sever	14(4.7)	19.50 (4.2)			11.86 (5.7)		
	Very sever	9(3.1)	18.78 (4.6)			15.33 (9.0)		

*ANOVA* Analysis of Variance

There were two exceptions in the relationship between GSI score and stress and anxiety. Therefore, Post Hoc Tukey’s test was used. According to Post Hoc Tukey’s test, the difference between the mean GSI scores in the group with severe anxiety and in the group with very severe anxiety was statistically significant (*p* = .024), but the difference in the mean GSI scores between the group with severe stress and other groups was not statistically significant (*p* > 0.05).

### The factors influencing maternal depression, anxiety and stress during pregnancy

The results of univariate and multivariate linear regression regarding the association between the related variables and depression, anxiety and stress are presented in Tables 4, 5, and 6, respectively.

**Table 4:** Univariate and multivariate linear regression examining related factors of depression among Iranian pregnant women (*N* = 295)

Participant characteristics	Univariate linear regression			Multivariate linear regression		
	Unstandard/ β	Standard/ Beta	*p*-value	Unstandard/ β	Standard/ Beta	*p*-value
**Age(y)**	0.330	0.235	< 0.001	0.307	0.218	0.001
**Duration of marriage(y)**	0.291	0.187	0.001	−0.154	−0.099	0.152
**Education level**	–	–	–			
Primary/secondary school				NI	NI	NI
High school	1.136	0.765	0.439			
Undergraduate/postgraduate	0.782	0.051	0.603			
**Women occupation**	–	–	–	–	–	–
Working						
Housewife	1.731	0.083	0.153	1.601	0.077	0.134
**Satisfaction with income**	–	–	–	–	–	–
Low						
Moderate	−4.715	−0.286	< 0.001	−2.020	− 0.122	0.023
High	−7.054	− 0.172	0.004	−2.747	−0.067	0.197
**Parity**	–	–	–	–	–	–
Primiparous						
Multiparous	2.903	0.196	0.001	1.655	0.111	0.064
**Planned pregnancy**	–	–	–	–	–	–
No						
Yes	−3.523	−0.203	< 0.001	−2.232	−0.128	0.014
**History of abortion**	–	–	–			
No				NI	NI	NI
Yes	0.676	0.038	0.517			
**Complication in previous pregnancy**	–	–	–	–	–	–
No						
Yes	5.199	0.219	< 0.001	2.410	0.101	0.053
**Fear of fetal abortion**	–	–	–	–	–	–
No						
Yes	3.175	0.251	< 0.001	1.473	0.099	0.070
**Gestational age**	−0.076	−0.094	0.107	−0.099	−0.122	0.018
**Satisfaction with BI in pregnancy**	–	–	–	–	–	–
Low						
Moderate	−3.429	−0.229	< 0.001	−2.176	−0.145	0.014
High	−5.202	−0.209	0.002	−2.189	−0.088	0.120
**Satisfaction with foreplay**	–	–	–	–	–	–
No						
Yes	−3.553	−0.141	0.015	− 0.446	−0.017	0.731
**Sexual distress**	0.483	0.429	< 0.001	0.322	0.285	< 0.001
**Genital Self-Image**	−0.435	−0.234	< 0.001	− 0.221	−0.118	0.023

*NI* Not Included in the multivariate linear regression

**Table 5:** Univariate and multivariate linear regression examining related factors of anxiety among Iranian pregnant women (*N* = 295)

Participant characteristics	Univariate linear regression			Multivariate linear regression		
	Unstandard/ β	Standard/ Beta	*p*-value	Unstandard/ β	Standard/ Beta	*p*-value
**Age(y)**	0.261	0.181	0.002	0.246	0.170	0.013
**Duration of marriage(y)**	0.313	0.196	0.001	0.054	0.033	0.641
**Education level**	–	–	–	NI	NI	NI
Primary/secondary school						
High school	−1.322	−0.089	0.384			
Undergraduate/postgraduate	−1.230	−0.078	0.426			
**Women occupation**	–	–	–	–	–	–
Working						
Housewife	2.847	0.133	0.022	2.655	0.124	0.021
**Satisfaction with income**	–	–	–	–	–	–
Low						
Moderate	−4.589	−.271	< 0.001	−2.605	−0.154	0.007
High	−7.361	−.175	0.003	−3.382	− 0.080	0.140
**Parity**	–	–	–	–	–	–
Primiparous						
Multiparous	1.685	0.111	0.058	0.220	0.014	0.818
**Planned pregnancy**	–	–	–	–	–	–
No						
Yes	−2.455	1.137	0.018	−1.099	−0.061	0.256
**History of abortion**	–	–	–			
No				NI	NI	NI
Yes	−0.057	− 0.003	0.958			
**Complication in previous pregnancy**	–	–	–	–	–	–
No						
Yes	3.981	0.163	0.005	1.111	0.045	0.406
**Fear of fetal abortion**	–	–	–	–	–	–
No						
Yes	4.314	0.284	< 0.001	3.149	0.207	< 0.001
**Gestational age**	−0.147	−0.176	0.002	− 0.144	− 0.172	0.001
**Satisfaction with BI in pregnancy**	–	–	–	–	–	–
Low						
Moderate	−2.729	−0.177	0.005	−2.890	− 0.187	0.003
High	−3.682	−0.144	0.022	− 2.214	−0.086	0.143
**Satisfaction with foreplay**	–	–	–	–	–	–
No						
Yes	−1.800	−0.069	0.235	1.036	0.039	0.458
**Sexual distress**	0.433	0.373	< 0.001	0.237	0.204	< 0.001
**Genital Self-Image**	−0.167	−0.087	0.134	0.030	0.016	0.767

*NI* Not Included in the multivariate linear regression

**Table 6:** Univariate and multivariate linear regression examining related factors of stress among Iranian pregnant women (*N* = 295)

Participant characteristics	Univariate linear regression			Multivariate linear regression		
	Unstandard/ β	Standard/ Beta	*p*-value	Unstandard/ β	Standard/ Beta	*p*-value
**Age(y)**	0.384	0.229	< 0.001	0.233	0.139	0.028
**Duration of marriage(y)**	0.409	0.220	< 0.001	0.113	0.061	0.368
**Education level**	–	–	–			
Primary/secondary school				NI	NI	NI
High school	1.055	0.059	0.546			
Undergraduate/postgraduate	1.850	0.102	0.302			
**Women occupation**	–	–	–			
Working				NI	NI	NI
Housewife	0.682	0.028	0.637			
**Satisfaction with income**	–	–	–	–	–	–
Low						
Moderate	−4.360	−0.222	< 0.001	−1.361	−0.069	0.197
High	−7.720	−0.158	0.008	−3.895	−0.080	0.122
**Parity**	–	–	–	–	–	–
Primiparous						
Multiparous	1.584	0.090	0.124	−1.110	−0.062	0.296
**Planned pregnancy**	–	–	–	–	–	–
No						
Yes	−4.406	−0.213	< 0.001	−4.033	−0.194	< 0.001
**History of abortion**	–	–	–			
No				NI	NI	NI
Yes	−0.260	−0.012	0.835			
**Complication in previous pregnancy**	–	–	–	–	–	–
No						
Yes	6.940	0.245	< 0.001	4.546	0.160	0.002
**Fear of fetal abortion**	–	–	–	–	–	–
No						
Yes	4.134	0.235	< 0.001	1.609	0.091	0.097
**Gestational age**	−0.144	−0.148	0.011	−0.120	−0.124	0.016
**Satisfaction with BI in pregnancy**	–	–	–	–	–	–
Low						
Moderate	−2.830	−0.158	0.012	−1.624	−0.091	0.124
High	−3.985	−0.134	0.032	−0.669	−0.022	0.688
**Satisfaction with foreplay**	–	–	–	–	–	–
No						
Yes	−3.492	−0.116	0.046	−0.934	− 0.31	0.546
**Sexual distress**	0.628	0.467	< 0.001	0.467	0.347	< 0.001
**Genital Self-Image**	−0.273	− 0.123	0.034	− 0.032	−0.014	0.782

*NI* Not Included in the multivariate linear regression

Women’s level of education and history of abortion were not significantly associated with antenatal depression and anxiety in the univariate linear regression analysis (*p* <  0.25), so they were not included in the multivariate linear regression analysis. According to multivariate linear regression analysis, a higher (worse) depression score was seen in women with more advanced age (β = 0.218, *p* = 0.001, i.e., 0.218 points higher for each unit higher women’s age) and among women with greater SD scores ([β = 0.285, *p* <  0.001, i.e., 0.285 points higher for each unit higher SD score). Depression score was associated with GSI score (β = − 0.118, *p* = 0.023), planned pregnancy (β = − 0.128, *p* = 0.014), satisfaction with income (β = − 0.122, *p* = 0.023), and satisfaction with BI in pregnancy (β = − 0.145, *p* = 0.014) in multivariate linear regression analysis, such that lower (better) depression scores were observed among women with higher (better) GSI score, women with planned pregnancy, moderate satisfaction with income, and moderate satisfaction with BI in pregnancy (Table 4).

Regarding anxiety, the following influencing factors were found to be statistically significant in multivariate linear regression analysis: SD score (β = 0.204, *p* <  0.001), fear of fetal abortion (β = 0.207, *p* <  0.001) women’s age (β = 0.170, *p* = 0.013), women’s occupation (β = 0.124, *p* = 0.021), gestational age (β = − 0.172, *p* = 0.001), moderate satisfaction with income (β = − 0.154, *p* = 0.007), moderate satisfaction with BI in pregnancy (β = − 0.187, *p* = 0.003), such that higher (worse) anxiety score was obtained by women with higher (worse) SD scores, women with fear of fetal abortion, housewives, and women with more advanced age; in addition, lower (better) anxiety score was noted among women with increased gestational age and those with moderate satisfaction with income and moderate satisfaction with BI in pregnancy (Table 5).

In the univariate linear regression analysis of antenatal stress, the variables that were not significantly associated with stress score included women’s occupation, women’s education level, and history of abortion. Finally, the significant factors influencing stress during pregnancy in multivariate linear regression analysis were SD (β = 0.347, *p* <  0.001), women’s age (β = 0.139, *p* = 0.028), complications in previous pregnancy (β = 0.160, *p* = 0.002), planned pregnancy (β = − 0.194, *p* <  0.001), and gestational age (β = − 0.124, *p* = 0.016), such that higher (worse) stress scores were found among women with higher (worse) SD scores, more advanced age, and experience of complications in previous pregnancies; besides, lower (better) stress scores were obtained by women with increased gestational age and those with planned pregnancy (Table 6).

## Discussion

The present study demonstrated that female SD was a significant factor contributing to depression, anxiety, and stress. Moreover, increased severity of SD in pregnancy was associated with elevated severity of depression, anxiety, and stress. Although many studies have examined sexual function in pregnancy, few have examined SD during this period. In addition, no study has yet been conducted to investigate this factor as an influential factor in depression, anxiety, and stress during pregnancy. A study of non-pregnant samples showed a significant relationship between anxiety and stress and all aspects of sexual function, except for sexual desire and pain. It also revealed a significant association between depression and all aspects of sexual function except for sexual pain [28]. In a recent study comparing female sexual dysfunction between groups of pregnant and non-pregnant women revealed a significantly higher sexual dysfunction in the pregnant group. Since women with sexual problems may report SD concurrently [36], it seems that SD to be also associated with anxiety, depression, and stress [37]. A study by Sarah also showed that 42% of women experience SD during pregnancy, which can be in the presence or absence of a sexual problem [13]. This rate of SD is slightly higher than the rate reported in Finnish and American population-based studies of women who were not pregnant [36, 38]. It seems that SD is more common in pregnancy due to the unique features of this period. For this reason, it is important to consider the role of SD in the development of depression, anxiety, and stress, followed by providing counseling and treatment techniques to ameliorate it.

We found that women with higher (better) GSI score were at a lower risk for depression in multivariate linear regression analysis. Indeed, we noted a significant inverse correlation between GSI score and depression and anxiety scores in ANOVA, such that as GSI score increased, the scores of depression and anxiety decreased. In other words, having a poor GSI was associated with high severity of depression and anxiety. In this regard, moderate satisfaction with BI in pregnancy was also recognized as an effective factor in antenatal anxiety and depression. In a critical review of the literature, it was reported that all prospective cohort studies evaluating the impact of BI on the incidence of depression found a positive relationship between BI dissatisfaction and incidence of prenatal depression. Moreover, this review examined whether depression leads to BI dissatisfaction. It was noted that the findings of studies are inconsistent regarding the effect of depression on BI [19]. As it is known, pregnancy induces a variety of hormonal, immunologic, and metabolic changes that exert significant effects on a woman’s body. Pregnancy can trigger or intensify negative feelings about the body. Some women can be distressed by bodily changes in pregnancy. BI dissatisfaction during pregnancy can have a negative impact on both the mother and the baby [39]. One of the areas most affected by pregnancy changes is the genitals (i.e., hyperpigmentation, volva skin stretch, striae on volva, mucosal changes, etc.) [18]. Both BI dissatisfaction and poor GSI can indirectly cause stress and anxiety and affect sexual function [40, 41], and subsequently, engender SD. Many studies have reported a link between GSI and sexual function in the non-pregnant population. Since pregnant women are more prone to poor GSI due to changes in their physiology, special attention should be paid to the role of this factor in the development of mental disorders.

Planned pregnancy was revealed to be a significant factor for developing depression and stress among participants in the present study. This finding has been corroborated by another study indicating the negative effect of unplanned pregnancy on antenatal depression. Biratu et al. [42] reported that women who had not planned the current pregnancy were 2.58 times more likely to develop antenatal depression than those who had planned the pregnancy. Many studies have reported unplanned or unwanted pregnancy as a risk factor for developing depression [42–44] and stress [45]. Various studies have emphasized the role of planned pregnancy in the prevention of antenatal depression [42]. In a systematic review conducted by Alessandra et al. [46], it was shown that unplanned or unwanted pregnancy is associated with antenatal depression. Hartley et al. [47] did not find a significant association between unwanted pregnancy and antenatal depression. Unplanned pregnancy can make a pregnant woman unhappy. Unhappiness could be the result of many contributing factors such as conflicts with others, life stress, and lack of social support. Sherin et al. [48] pointed out that being unhappy with the pregnancy is another risk factor associated with antenatal depression and women who were unhappy with their pregnancy were more likely to suffer from antenatal depression.

This study demonstrated that the presence of complications in previous pregnancies (for any reason) was associated with stress in the current pregnancy. Negative obstetric history such as history of miscarriage, stillbirth, and preterm birth and other pregnancy complications like hyperemesis gravidarum, hypertension, and diabetes mellitus could pose additional stress on mothers in current pregnancy [49]. These reports show the importance of asking all pregnant women, regardless of their cultural differences, about history of experiencing any bad events for the mother or her fetus in a previous pregnancy.

We also found that fear of fetal abortion was a significant risk factor for anxiety, which it could be due to lack of experience in becoming a mother in primiparous women or due to bad experiences and complications in previous pregnancies in multiparous women. Therefore, it is necessary to ask about the fears and worries of pregnant women in prenatal care and provide appropriate counseling to reduce these negative feelings.

Moderate satisfaction with income was another factor closely related to the decreased risk of prenatal depression and anxiety in this study, which is consistent with the results of other studies. For example, in a study by Brittain et al. [43] in South Africa, it was found that pregnant women with low socioeconomic status were more likely to experience antenatal depression than those with a high socioeconomic status. Another study also suggested that the higher prevalence of depression among black and Hispanic mothers could be mainly due to lower income and financial problems [3]. These findings were also supported by a recent systematic review by Fekadu Dadi et al. [50], which reported that pregnant women with a history of economic difficulties were more likely to report antenatal depression. Nevertheless, family income in a study conducted in Malaysia was not associated with depression and anxiety in pregnancy [51]. This discrepancy could be due to difference in study populations and measuring income levels using different categories.

The present study also demonstrated a statistically significant association between the presence of antenatal anxiety and women’s occupation, evidencing that housewives had a greater chance of developing anxiety during pregnancy than working women. This finding is similar to that found by a researcher in a study conducted in Brazil [52]. However, contrary to these findings, a study on the prevalence and factors associated with anxiety during pregnancy in Italy did not show a significant relationship between employment status (such as student, housewife, unemployed, employed, manager) and prenatal anxiety [53]. A possible explanation for incompatible findings may be differences in the categories considered for women’s occupation. Housewives are less busy and have more free time to think about pregnancy and its consequences, so they may experience higher levels of anxiety. In addition, they may perceive more anxiety due to their financial dependence on their partner for the new role of motherhood.

Our findings support the significant role of gestational age in the development of depression, anxiety, and stress, such that increased gestational age was associated with a decrease in their scores. Fadzil et al. [51] showed that gestational age less than 20 weeks was associated with antenatal anxiety. The risk of antenatal anxiety disorder was 4.85 times higher among mothers below 20 weeks of gestation compared to those 20 weeks and above. The probable reason for this finding is the increase in a woman’s ability to cope with the physiological process of pregnancy and the physical changes occurring in her body over time. What’s more, increased age of women was revealed to be a significant risk factor for depression, anxiety and stress in current study, which could be because aging is associated with more pregnancy complications such as preeclampsia and worry about having these complications, which may lead to higher stress and anxiety in women.

In summary, this study provides crucial information as to the factors related to depression, anxiety, and stress during pregnancy among Iranian women. Indeed, this is the first study to consider SD and GSI as related factors that can affect depression, anxiety, and stress in pregnancy. It is important to be aware of the factors contributing to the development of antenatal depression, anxiety, and stress, pay attention to these factors in prenatal care, and offer counseling strategies to reduce the depression, anxiety, and stress perceived by pregnant women based on the factors causing them.

## Conclusions

This study demonstrates the necessity for psychiatric counseling based on predisposing factors for promoting the mental health of pregnant women and preventing adverse effects for the fetus in pregnancy. Therefore, it is recommended that midwives and nurses evaluate pregnant women for these risk factors and provide preventive care and, if necessary, refer pregnant women to a psychiatrist for treatment of the severe forms of these disorders.

Some limitations of the study should be noted. First of all, caution should be exercised in the interpretation of the data as the cross-sectional nature of the study does not show the causal relationship of the variables and correlational and longitudinal studies are needed to be conducted in the future. Although this study attempted to comprehensively examine the factors associated with mental disorders, some factors such as social support, satisfaction with the partner, and experience of domestic violence were not evaluated. Moreover, previous studies suggest that prenatal mental disorders often coexist and affect each other, but our study disregarded the role of one mental factor on another.

## Supplementary Information

**Additional file 1.** Socio-demographics Checklist

## Data Availability

The datasets for current study are available from the corresponding author on reasonable request.

## Abbreviations

SD: Sexual Distress
GSI: Genital Self-Image
BI: Body Image
DASS-21: Depression Anxiety and Stress Scale-21
SIDI-F: Sexual Interest and Desire Inventory-Female
FSDS-R: Female Sexual Distress Scale-Revised
STROBE: STrengthening the Reporting of OBservationally studies in Epidemiology
ANOVA: Analysis of Variance
NI: Not Included

## References

[1] Senobari M, Azmoude E, Mousavi M (2019). The relationship between body mass index, body image, and sexual function: a survey on Iranian pregnant women. Int J Reprod BioMed.

[2] Carter D, Kostaras X (2005). Psychiatric disorders in pregnancy. British Columbia Med J.

[3] Rich-Edwards JW, Kleinman K, Abrams A, Harlow BL, McLaughlin TJ, Joffe H, Gillman MW (2006). Sociodemographic predictors of antenatal and postpartum depressive symptoms among women in a medical group practice. J Epidemiol Community Health.

[4] Pereira PK, Lovisi GM, Pilowsky DL, Lima LA, Legay LF (2009). Depression during pregnancy: prevalence and risk factors among women attending a public health clinic in Rio de Janeiro, Brazil. Cadernos de Saúde Pública.

[5] Tang X, Lu Z, Hu D, Zhong X (2019). Influencing factors for prenatal stress, anxiety and depression in early pregnancy among women in Chongqing, China. J Affect Disord.

[6] Zareipour M, Sadaghianifar A, Amirzehni J, Parsnezhad M, Ayuoghi Rahnema V (2017). Exploring of depression, anxiety and stress in pregnant women referred to health centers of Urmia. Rahavard Salamat J.

[7] Kaplan LA, Evans L, Monk C (2008). Effects of mothers’ prenatal psychiatric status and postnatal caregiving on infant biobehavioral regulation: can prenatal programming be modified?. Early Hum Dev.

[8] DeRogatis L, Clayton A, Lewis-D’Agostino D, Wunderlich G, Fu Y (2008). Validation of the female sexual distress scale-revised for assessing distress in women with hypoactive sexual desire disorder. J Sex Med.

[9] Bartellas E, Crane JM, Daley M, Bennett KA, Hutchens D (2000). Sexuality and sexual activity in pregnancy. BJOG.

[10] Pauls RN, Occhino JA, Dryfhout VL (2008). Effects of pregnancy on female sexual function and body image: a prospective study. J Sex Med.

[11] Skouteris H, Carr R, Wertheim EH, Paxton SJ, Duncombe D (2005). A prospective study of factors that lead to body dissatisfaction during pregnancy. Body Image.

[12] Bailey L (2001). Gender shows: first-time mothers and embodied selves. Gend Soc.

[13] Vannier SA, Rosen NO (2017). Sexual distress and sexual problems during pregnancy: associations with sexual and relationship satisfaction. J Sex Med.

[14] DeMaria AL, Meier SJ, Dykstra C (2019). “It’s not perfect but it’s mine”: genital self-image among women living in Italy. Body Image.

[15] Baršauskaitė I (2018). Female genital self-image and sexual dissatisfaction: the role of sexual esteem and partner perceived genital dissatisfaction.

[16] Inanir S, Cakmak B, Nacar MC, Guler AE, Inanir A (2015). Body image perception and self-esteem during pregnancy.

[17] Earle S. “bumps and boobs”: fatness and women’s experiences of pregnancy. Women’s Stud Int Forum. 2003;26(3):245–52.

[18] Motosko CC, Bieber AK, Pomeranz MK, Stein JA, Martires KJ (2017). Physiologic changes of pregnancy: a review of the literature. Int J Womens Dermatol.

[19] Silveira ML, Ertel KA, Dole N, Chasan-Taber L (2015). The role of body image in prenatal and postpartum depression: a critical review of the literature. Arch Womens Mental Health.

[20] Roomruangwong C, Kanchanatawan B, Sirivichayakul S, Maes M (2017). High incidence of body image dissatisfaction in pregnancy and the postnatal period: associations with depression, anxiety, body mass index and weight gain during pregnancy. Sex Reprod Healthc.

[21] Sweeney AC, Fingerhut R (2013). Examining relationships between body dissatisfaction, maladaptive perfectionism, and postpartum depression symptoms. J Obstet Gynecol Neonatal Nurs.

[22] Von Elm E, Altman DG, Egger M, Pocock SJ, Gøtzsche PC, Vandenbroucke JP (2007). The Strengthening the reporting of observational studies in epidemiology (STROBE) statement: guidelines for reporting observational studies. Ann Intern Med.

[23] Nik-Azin A, Nainian MR, Zamani M, Bavojdan MR, Bavojdan MR, Motlagh MJ (2013). Evaluation of sexual function, quality of life, and mental and physical health in pregnant women. J Family Reprod Health.

[24] Lovibond PF, Lovibond SH (1995). The structure of negative emotional states: comparison of the depression anxiety stress scales (DASS) with the Beck depression and anxiety inventories. Behav Res Ther.

[25] Oei TP, Sawang S, Goh YW, Mukhtar F (2013). Using the depression anxiety stress scale 21 (DASS-21) across cultures. Int J Psychol.

[26] Crawford JR, Henry JD (2003). The depression anxiety stress scales (DASS): normative data and latent structure in a large non-clinical sample. Br J Clin Psychol.

[27] Moradipanah F, Mohammadi E, Mohammadil A (2005). The effect of music on anxiety stress and mild depression patients undergoing cardiac catheterization [Dissertation].

[28] Yazdanpanahi Z, Beygi Z, Akbarzadeh M, Zare N (2018). To investigate the relationship between stress, anxiety and depression with sexual function and its domains in women of reproductive age. Int J Med Res Health Sci.

[29] Nowosielski K, Wróbel B, Sioma-Markowska U, Poręba R (2013). Sexual dysfunction and distress—development of a polish version of the female sexual distress scale-revised. J Sex Med.

[30] Nekoo EA, Burri A, Ashrafti F, Fridlund B, Koenig HG, Derogatis LR, Pakpour AH (2014). Psychometric properties of the Iranian version of the female sexual distress scale-revised in women. J Sex Med.

[31] Aydın S, Onaran ÖI, Topalan K, Aydın ÇA, Dansuk R (2016). Development and validation of Turkish version of the female sexual distress scale-revised. Sex Med.

[32] Herbenick D, Reece M (2010). Outcomes assessment: development and validation of the female genital self-image scale. J Sex Med.

[33] Herbenick D, Reece M (2010). Development and validation of the female genital self-image scale. J Sex Med.

[34] Felix GD, Nahas FX, Marcondes GB, dos Santos AG, de Brito MJ, Ferreira LM (2017). Brazilian Portuguese version of the female genital self image scale (FGSIS) for women seeking abdominoplasty. J Plast Reconstr Aesthet Surg.

[35] Pakpour AH, Zeidi IM, Ziaeiha M, Burri A (2014). Cross-cultural adaptation of the female genital self-image scale (FGSIS) in Iranian female college students. J Sex Res.

[36] Shifren JL, Monz BU, Russo PA, Segreti A, Johannes CB (2008). Sexual problems and distress in United States women: prevalence and correlates. Obstet Gynecol.

[37] Anğın AD, Özkaya E, Çetin M, Gün I, Sakin O, Ertekin LT, Denizli R, Koyuncu K, Akalın EE (2020). Comparison of female sexual function and sexual function of their partners between groups of pregnant and non-pregnant women. Ginekol Pol.

[38] Witting K, Santtila P, Varjonen M, Jern P, Johansson A, Von Der Pahlen B, Sandnabba K (2008). Female sexual dysfunction, sexual distress, and compatibility with partner. J Sex Med.

[39] Brown A, Rance J, Warren L (2015). Body image concerns during pregnancy are associated with a shorter breast feeding duration. Midwifery.

[40] Lordelo P, Brasil C, Lerche J, Gomes T, Martins P, Castro M (2017). Relationship between female genital self-image and sexual function: cross-sectional study. Obstet Gynecol Int J.

[41] Jawed-Wessel S, Herbenick D, Schick V (2017). The relationship between body image, female genital self-image, and sexual function among first-time mothers. J Sex Marital Ther.

[42] Biratu A, Haile D (2015). Prevalence of antenatal depression and associated factors among pregnant women in Addis Ababa, Ethiopia: a cross-sectional study. Reprod Health.

[43] Brittain K, Myer L, Koen N, Koopowitz S, Donald KA, Barnett W, Zar HJ, Stein DJ (2015). Risk factors for antenatal depression and associations with infant birth outcomes: results from a S outh a frican birth cohort study. Paediatr Perinat Epidemiol.

[44] Karaçam Z, Ançel G (2009). Depression, anxiety and influencing factors in pregnancy: a study in a Turkish population. Midwifery.

[45] Nagandla K, Nalliah S, Yin L, Majeed Z, Ismail M, Zubaidah S, Ragavan U, Krishnan S (2016). Prevalence and associated risk factors of depression, anxiety and stress in pregnancy. Int J Reprod Contracept Obstet Gynecol.

[46] Biaggi A, Conroy S, Pawlby S, Pariante CM (2016). Identifying the women at risk of antenatal anxiety and depression: a systematic review. J Affect Disord.

[47] Hartley M, Tomlinson M, Greco E, Comulada WS, Stewart J, Le Roux I, Mbewu N, Rotheram-Borus MJ (2011). Depressed mood in pregnancy: prevalence and correlates in two Cape Town peri-urban settlements. Reprod Health.

[48] Mohamad Yusuff AS, Tang L, Binns CW, Lee AH (2016). Prevalence of antenatal depressive symptoms among women in Sabah, Malaysia. J Matern Fetal Neonatal Med.

[49] Chou F-H, Kuo S-H, Wang R-H (2008). A longitudinal study of nausea and vomiting, fatigue and perceived stress in, and social support for, pregnant women through the three trimesters. Kaohsiung J Med Sci.

[50] Fekadu Dadi A, Miller ER, Mwanri L (2020). Antenatal depression and its association with adverse birth outcomes in low and middle-income countries: a systematic review and meta-analysis. PLoS One.

[51] Fadzil A, Balakrishnan K, Razali R, Sidi H, Malapan T, Japaraj RP, Midin M, Nik Jaafar NR, Das S, Manaf MRA (2013). Risk factors for depression and anxiety among pregnant women in Hospital Tuanku Bainun, Ipoh, Malaysia. Asia Pac Psychiatry.

[52] Silva MMdJ, Nogueira DA, Clapis MJ, Leite EPRC: Anxiety in pregnancy: prevalence and associated factors. Revista da Escola de Enfermagem da USP. 2017;51:e03253. 10.1590/S1980-220X2016048003253 28902327

[53] Giardinelli L, Innocenti A, Benni L, Stefanini M, Lino G, Lunardi C, Svelto V, Afshar S, Bovani R, Castellini G (2012). Depression and anxiety in perinatal period: prevalence and risk factors in an Italian sample. Arch Womens Mental Health.

