# Supplementary material for: Factors influencing stress, anxiety, and depression among Iranian pregnant women: the role of sexual distress and genital self-image
Source: BMC Pregnancy Childbirth. 2021 Jan 26;21:87. doi: 10.1186/s12884-021-03575-1 (PMC7836496; doi:10.1186/s12884-021-03575-1)
Supplement: Supplementary file 1 — Additional file 1. Socio-demographics Checklist [file 12884_2021_3575_MOESM1_ESM.docx]

**Socio-demographics Checklist**

The socio-demographics checklist was composed of objective questions designed by the researchers and contained socio-demographic (i.e., age, duration of marriage, women’s education, women’s occupation, satisfaction with income) and obstetric (parity, planned pregnancy, history of abortion, complications in previous pregnancy, gestational age, and fear of fetal abortion) questions. It also asked about satisfaction with foreplay and BI.

The questions in this checklist include the following:

Your age: ……

How long has it been since your marriage?.......

What is your education level?

Primary/secondary school

High school

Undergraduate/postgraduate

What is your occupation?

Working

Housewife

How satisfied are you with your income level? Low Moderate High

Your gestational age: ……

How many times have you been pregnant?......

Was your pregnancy planned? Yes No

Have you had a history of complications in your previous pregnancy? Yes No

Did you have a history of abortion? Yes No

Are you afraid of abortion in your current pregnancy? Yes No

How satisfied are you with body image in pregnancy? Low Moderate High

Are you satisfied with the foreplay? Yes No
